# Safety and Efficacy of Achilles Repair Using the Mini-Open Approach in Supine Versus Prone Position: A Retrospective Study

**DOI:** 10.7759/cureus.17564

**Published:** 2021-08-30

**Authors:** Haley McKissack, Ryan McLynn, Charles Pitts, Bradley Alexander, James Jones, Nicholas A Andrews, Zachary L Littlefield, Ashish Shah

**Affiliations:** 1 Department of Orthopaedic Surgery, University of Alabama at Birmingham, Birmingham, USA

**Keywords:** achilles rupture, achilles repair, mini-open approach, prone, supine

## Abstract

Purpose

Surgical repair of the Achilles tendon is a common procedure in cases of acute rupture. Open Achilles tendon surgery with a traditional extensile approach is most often performed in the prone position, but this can lead to numerous complications. The mini-open approach for repair in the supine position may avoid the risks of the prone position. The purpose of this study is to compare perioperative outcomes and differences in cost between patients undergoing acute Achilles rupture repair with mini-open approach, incision of approximately 3 cm, in the supine position versus traditional approach in the prone position.

Methods

Patients who underwent surgical repair of acute Achilles rupture at a single institution were retrospectively identified using Current Procedural Terminology (CPT) code 27650. Complication rates and the total cost charged to the insurance companies of both the supine and prone groups were calculated.

Results

A total of 80 patients were included for analysis, 26 supine and 54 prone. The difference in average total time in the operating room was statistically significant. The prone position took approximately 15% more time (118.7 minutes) compared to the supine position (100 minutes) (p = 0.001). While not statistically significant, the total cost for the supine group ($19,889) was less than the for the prone group ($21,722) (p = 0.153) Average postoperative pain score, infection rate, dehiscence rate, sepsis rate, and deep vein thrombosis (DVT) rate were also similar between the two groups. No patient in either group experienced re-rupture of the Achilles tendon within the first year of primary repair.

Conclusion

The mini-open approach in the supine position may be advantageous in the repair of acute Achilles rupture in that it reduces total time in the operating room and total cost while maintaining positive patient outcomes. Prospective clinical studies are warranted to validate these assessments.

## Introduction

Achilles tendon rupture is a growing problem with various options for conservative and operative management [1]. Surgical management has the advantage of having a lower incidence of re-rupture while allowing patients to return to work and other activities sooner than those treated nonoperatively [1,2]. Traditionally, surgical repair has been performed via an open approach in the prone position. While this approach achieves acceptable outcomes in terms of healing and return to activity, it has been associated with high incidence of wound complications and injury to the sural nerve [2-4]. As a result, less invasive approaches have been developed with the goal of achieving comparable repair while minimizing morbidities associated with surgery. The mini-open approach, characterized by Kakiuchi in 1995, allows repair under direct visualization with the goal of reducing surgical site complications [5]. Several studies have since described the mini-open approach, reporting sound biomechanical properties and excellent functional outcomes while also achieving a faster return to activity and lower incidence of wound complications compared to open techniques [6-8].

Prone positioning requires increased positioning time and greater difficulty in airway management [9]. These complexities can potentially increase surgical and perioperative times, which can contribute to increased costs. Prone positioning has also been associated with complications including dislodgment of the endotracheal tube, elevated intraabdominal pressures, postoperative vision loss, cardiovascular complications, and cranial nerve and brachial plexus palsies [10-12]. These complexities are even greater in obese patients, who already present with challenging airways and increased risk of postoperative infections and wound complications [13,14]. Since 2008, there have been several technique reports and case series of repairing acute Achilles tendon ruptures in the supine position [15-17]. While the authors have noted the ease of positioning and generally good outcomes, previous studies have generally utilized a larger incision more comparable to the traditional prone approach [15]. No study has offered a detailed description of the complications of supine positioning using the mini-open approach with comparison to prone positioning. Additionally, no studies have compared the healthcare costs between these two approaches.

A mini-open approach in the supine position offers the potential for comparable success and decreased morbidity of a mini-open approach while avoiding the pitfalls of prone surgery [18]. The current study aimed to compare the outcomes and costs between primary Achilles repair with a supine mini-open approach and a traditional prone approach.

## Materials and methods

Data collection

A retrospective chart review, with IRB approval (IRB-300000382 - "Outcomes of Foot & Ankle Surgery"), was conducted to compare perioperative characteristics and postoperative outcomes between patients undergoing Achilles tendon repair using the extensile approach in the prone position and the mini-open approach in the supine position. All patients who underwent surgical repair of acute Achilles rupture between the years 2011 and 2018 at a single institution were identified using Current Procedural Terminology (CPT) code 27650. Patients who underwent concurrent procedures for additional injuries were excluded. All surgeries at the institution were performed by two board-certified, fellowship-trained foot and ankle surgeons. One surgeon completed all of the mini-open approach surgeries in the supine position and one surgeon completed all of the extensile approach surgeries in the prone position. The same amount of operative staff was available for each case type.

A total of 80 patients met the inclusion criteria. Demographic information, intraoperative, perioperative information, and postoperative outcomes were collected for each patient (Tables 1-3). Additionally, surgery time, total time in the operating room, and Post-Anesthesia Care Unit (PACU) time were collected as documented in the patient’s operative and PACU reports. Surgical time was defined as the time from incision to complete closure, and time in the operating room was defined as the time of operating room entry until the time of operating room exit. PACU time was defined as the time from PACU entry until PACU discharge. Postoperative outcomes included postoperative complications and VAS pain scores at two weeks, six weeks, three months, and six months.

Cost was calculated for each patient, and the differences in average costs were compared between the prone and supine groups. In order to prevent the bias of insurance reimbursement, the total cost charged to the patient’s insurance company was compared between the two groups. Cost was defined as the sum of the surgeon’s fees, hospital charges, and operating room charges as recorded by the institution’s billing department.

Statistical analysis

Statistical analysis was performed using Stata SE 15.0 (StataCorp LLC, College Station, USA). P-values of less than 0.05 were considered significant.

Mini-open supine technique

At the study institution, the mini-open supine technique is performed as described by Cone et al [18]. With the patient in the supine position on a Skytron table (Skytron LLC, Grand Rapids, USA), lower extremity bolsters are placed beneath the patient’s contralateral hip and operative ankle to allow for exaggerated external rotation of the ankle and improved medial visualization. A tourniquet is then applied above the knee on the operative side with subsequent sterile preparation in a standard fashion. A safety strap across the waist is recommended to ensure stable patient positioning, with the contralateral lower extremity secured to the bed with foam padding and tape. Following exsanguination and tourniquet inflation, the procedure commences.

**Figure 1:**
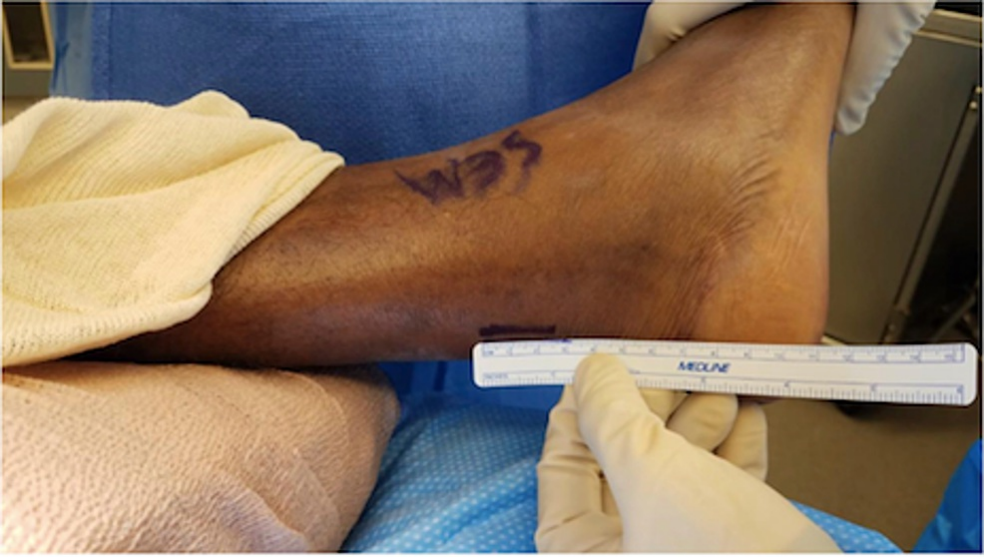
Measurement of incision line marked by a marking pen

**Figure 2:**
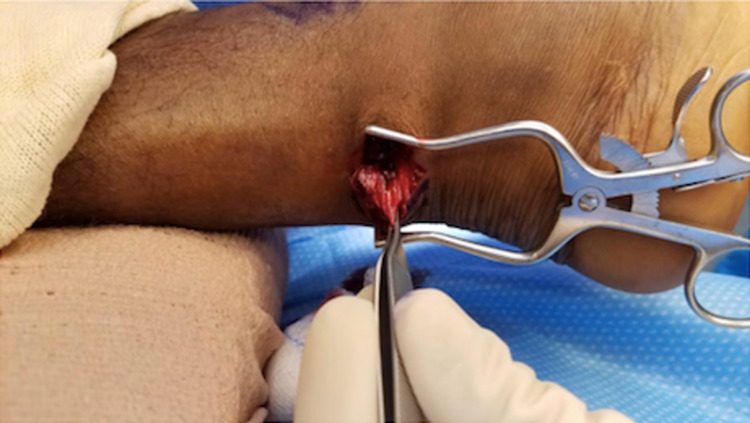
Subcutaneous tissues are spread to encounter the Achilles paratenon

The Achilles tendon defect is palpated and marked. A 3-4 cm longitudinal incision is made through the superficial layer, just medial to the midline of the tendon (Figure 1). The subcutaneous tissues are spread with scissors to encounter the Achilles paratenon (Figure 2). Scissors are then used to longitudinally release the paratenon, exposing the injury zone. The area is then cleared of debris and hematoma.

**Figure 3:**
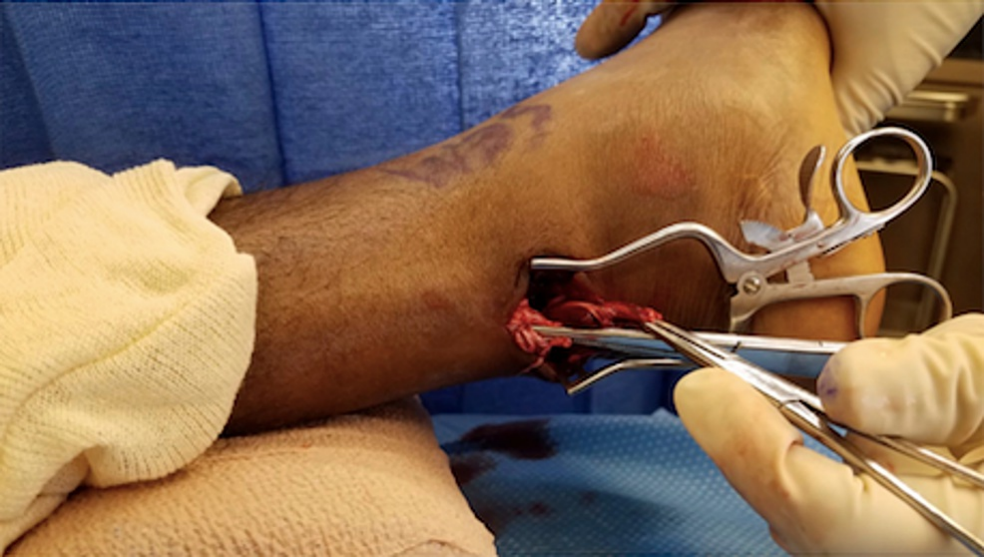
The ankle is plantarflexed, delivering the distal segment through the incision and into view

**Figure 4:**
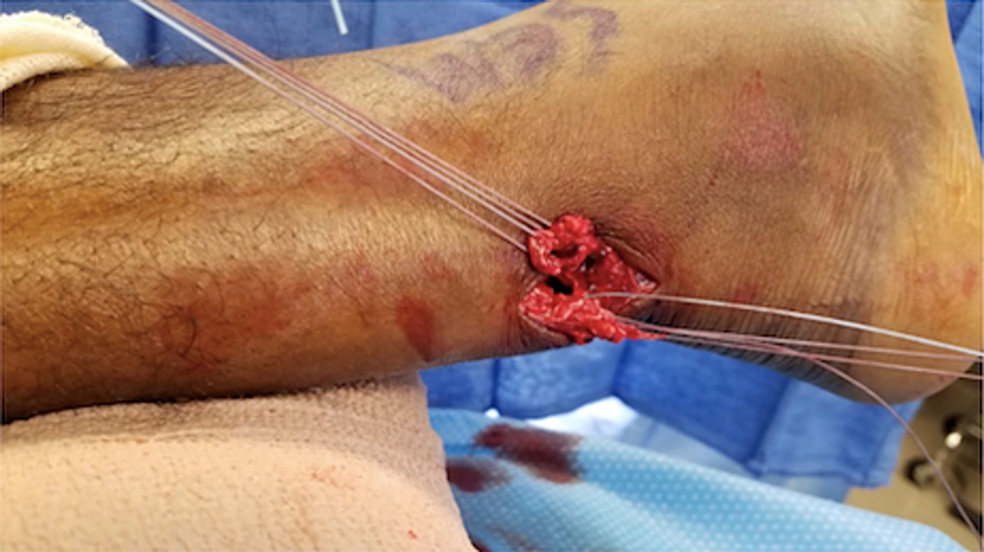
The four-stranded double Krackow locking stitch is demonstrated here after application to both the proximal tendon and distal tendon segments

Attention is first directed to the distal stump. The ankle is plantarflexed, delivering the distal segment through the incision and into view (Figure 3). Once the distal stump is exposed, we use #2 FiberWire (Arthrex, Inc, Naples, USA) for a four-stranded double Krackow locking stitch. This stitch uses one FiberWire to create two rows of locking stitches on the medial half of the tendon and another FiberWire to form two rows on the lateral half of the tendon. The technique should leave four free suture ends from the distal stump (Figure 4).

**Figure 5:**
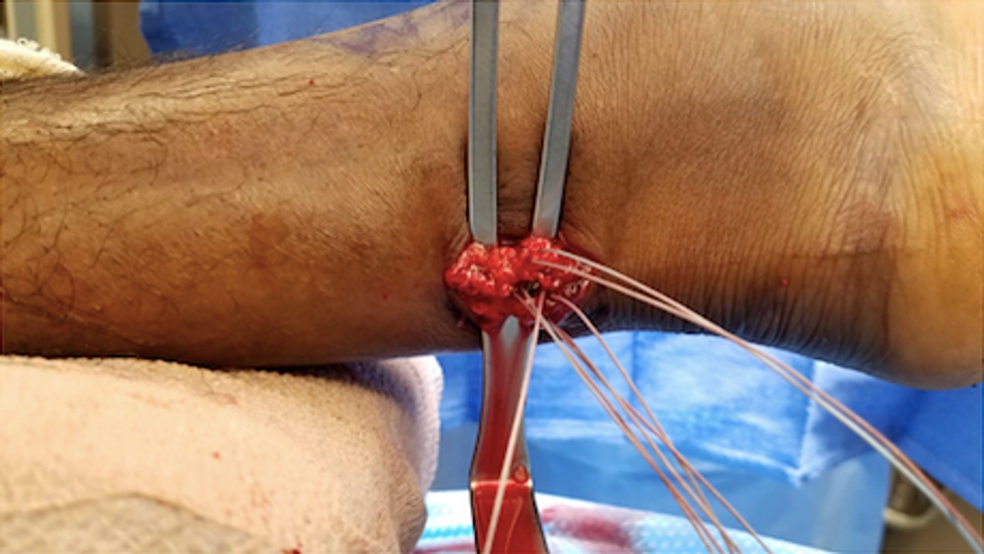
The free suture ends are shown paired and tied to approximate the ends of the ruptured tendon, such that tension of the injured side matches that of the uninjured side

Next, attention is moved to the proximal stump. Tenotomy scissors can be used to longitudinally incise more paratenon for improved visualization. With adequate exposure, the four-stranded double Krackow locking stitch is then applied to the proximal tendon. The free suture ends are then paired and tied to approximate the ends of the ruptured tendon, such that the repaired side is left under tension (Figure 5). This typically represents about 5 degrees of plantarflexion. Next, a 0 Vicryl (Ethicon, Inc., Bridgewater, USA) is used as a running the epitendinous suture for additional reinforcement.

**Figure 6:**
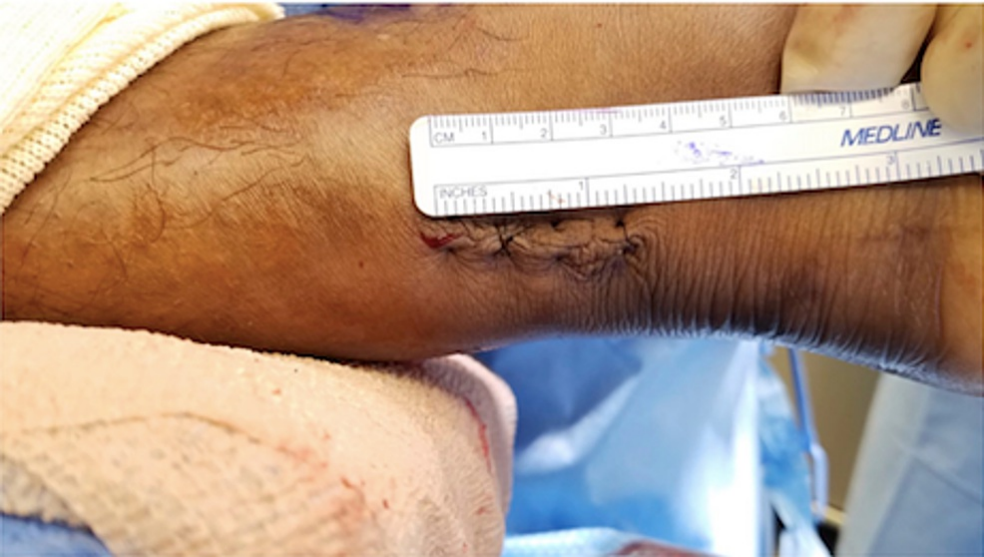
Subcutaneous tissue is closed with 4-0 Monocryl, followed by 3-0 nylon suture for the skin

The wound is irrigated with sterile saline and the paratenon is closed with a 2-0 Vicryl suture. Subcutaneous tissue is closed with 4-0 Monocryl (Ethicon, Inc., Bridgewater, USA), followed by 3-0 nylon suture for the skin (Figure 6). Postoperatively, a short leg-splint is applied with the ankle in slight plantarflexion.

## Results

A total of 80 patients met study criteria; 54 patients underwent traditional prone Achilles repair, while 26 underwent supine mini-open repair. The technique decided for each patient was based on the surgeon performing the procedure. One of the board-certified, fellowship-trained foot and ankle surgeons was only assigned supine and the other only prone, each chose based on their preference before starting the study. No significant differences were found between the two study groups at baseline as seen in Table 1.

**Table 1:** Patient demographics

		Supine Mini-open approach		Prone Traditional Extensile Approach		p-value
Average Age (years)		31.4		35.2		0.105
Sex						
Female		2		11		
Male		24		43		
Race (N)						
White		3		17		
Black		23		37		
Average BMI		27.2		28.4		0.369
Tobacco Use (N)		8		12		0.408
Substance use (N)		2		8		0.367

Table 2 shows the comparison of perioperative variables between the two patient groups as well as cost comparison. Average time spent in the operating room was longer for patients undergoing repair with the extensile approach in the prone position (118.7 minutes) compared to patients undergoing repair with the mini-open approach in the supine position (100 minutes, p = 0.001). Although not statistically significant (p = 0.153), the average total cost was higher in the traditional group ($21,722) compared to the supine mini-open group ($19,889).

**Table 2:** Operative variables PACU: post-anesthesia care unit

	Supine Mini-open approach	Prone Traditional Extensile Approach	p-value
Number of patients (N)	26	54	
Average time in operating room (minutes)	100	118.7	0.001
Average time in surgery (minutes)	62	68.5	0.150
Average tourniquet Time (minutes)	61	53.9	0.247
Average blood loss (mL)	4.6	6.3	0.248
Average incision length (cm)	4.4	5.8	0.163
Average time in PACU (minutes)	59	63.4	0.387
Average total cost (U.S. dollars)	19,889	21,722	0.153

The comparison of surgical outcomes is demonstrated in Table 3. One patient in the traditional approach group experienced superficial infection, there were no instances of infection in the supine mini-open group. Four patients in the prone group experienced wound dehiscence, of which three healed with conservative wound care and antibiotics. One required return to the operating room for debridement and secondary closure. One patient in the supine mini-open group experienced wound dehiscence, which healed with conservative wound care and antibiotics. Differences in the incidence of wound dehiscence between the groups were not statistically significant. No intraoperative complications in either group. No cases of deep infection, sepsis, or re-rupture of the tendon occurred in either group. Deep infection was defined as a patient needing debridement, IV antibiotics, and a peripherally inserted central catheter (PICC) line for the treatment of an infection. The average three-month visual analog scale (VAS) score of patients in the prone group was lower than those in the mini-open group, but by six months, both patient groups had similar average VAS scores although neither was statistically significant (p = 0.151; p = 0.907).

**Table 3:** Surgical outcomes DVT: deep vein thrombosis

	Supine Mini-open approach	Prone Traditional Extensile Approach	p-value
Average Pain score, 2 weeks	1.7	1.9	0.807
Average Pain score, 6 weeks	0.7	1.4	0.283
Average Pain score, 3 months	2.9	1.4	0.151
Average Pain score, 6 months	1	0.8	0.907
Superficial infection (N)	0	1 (1.9%)	0.444
Deep infection (N)	0	0	
Wound dehiscence (N)	1 (3.8%)	4 (7.4%)	0.424
Sepsis (N)	0	0	
DVT (N)	1 (3.8%)	0	0.185
Implant failure/removal (N)	0	0	
Re-Rupture (N)	0	0	

## Discussion

Traditional techniques to repair acute Achilles tendon ruptures expose patients to a relatively high incidence of surgical site complications as well as increased anesthesia time and potential complications associated with prone positioning. These factors can result in increased healthcare costs and resource utilization. Head-to-head studies comparing different surgical approaches have been limited and the quality of evidence has been relatively low [3]. This study found that a mini-open approach performed with the patient in the supine position was associated with significantly shortened time in the operating room, while still achieving comparable outcomes to those of patients undergoing repair in the prone position with the traditional approach.

The results of the current study demonstrated significantly reduced time in the operating room among patients undergoing Achilles repair in the supine position, a reduction that decreases resource utilization and healthcare dollar expenditures. The cost of anesthesia is directly related to the duration of the service and the personnel needed during the critical induction and reversal periods [19]. Simplifying induction and positioning, the supine position may decrease anesthesia time, translating to decreased costs for the patient. Additionally, while not directly billed for, operating room and personnel time are a major cost to hospitals. By shortening procedures and reducing the number of support staff needed to assist in prone positioning, supine positioning provides an avenue for hospitals to reduce costs while allowing physicians to dedicate their time to other responsibilities. Minimization of cost and resource usage is particularly important in the era of bundled payments, in which a set reimbursement is made to providers per specific service. Thus, as more resources are used, reimbursement for each physician within the bundled plan decreases.

Patients who underwent the prone approach spent approximately 19 more minutes in the operating room compared to patients in the supine position. This difference largely represents time dedicated to positioning after induction and in flipping the patient after the case. In addition to increasing time in the operating room, prone positioning subjects patients to a variety of potential complications [19]. Careful coordination in maintaining appropriate alignment of the neck and spine is important to avoid the possible complication of ischemic strokes [20]. This is particularly true among patients with undiagnosed carotid or vertebral artery stenosis, resulting in disrupted blood flow through the vertebral and carotid arteries [20-22]. Cervical spine injuries have also been reported secondary to excessive neck flexion or extension [23,24]. There is also the risk of dislodgment, kinking, and obstruction of the endotracheal tube as the patient is moved to the prone position [25]. We did not record any complications related to prone positioning in our series.

There are also complications associated with the patient remaining in the prone position throughout the surgery. Direct pressure on the face may lead to vision loss secondary to ischemic neuropathy or retinal artery occlusion [26]. Although rare, cardiovascular complications are of concern due to decreased cardiac index in the prone position. If any serious cardiovascular event were to occur, prone positioning can impede the placement of defibrillator pads [27]. Chest rolls used to provide anterior support to the patient have been proposed to increase venous pressure within the abdomen and obstruct the inferior vena cava, contributing to increased blood loss and decreased perfusion to the spinal cord, which can lead to paralysis [19]. Respiratory compromise is also a concern due to compression of the endotracheal tube during prone positioning. This risk is notable among those with spinal deformities [27]. Patients may also be at risk for neurological injury as both excessive extension and abduction of the shoulder have resulted in brachial plexus injury [28,29]. Anterior shoulder dislocations and postoperative shoulder pain have been described secondary to arms placed in extension above the head [19]. In some prone positioning techniques, the hips and knees are flexed (the “Tuck” position), which can impair peripheral circulation and cause muscle ischemia. In these instances, potential compartment syndrome and rhabdomyolysis are of concern [19,30].

The overall incidence of complications was demonstrated to be lower among the supine mini-open approach group in comparison to the prone extensile approach group, with rates of 7.7% and 9.3%, respectively. A study by Marcel et al. (2018) showed comparable results and, similar to our study, demonstrated zero incidences of re-rupture or sural neuritis at median follow-up of 116 days [17]. However, this study did not compare the outcomes and cost-effectiveness to individuals undergoing surgery with the prone approach. The results of the current study demonstrate that repair with the supine mini-open approach is at least as safe as repair with the prone approach, and trends may approach statistical significance with larger sample sizes.

When assessing wound complications specifically, the current study demonstrated that 9.3% of patients treated with the prone extensile approach, experienced wound complications. This incidence was consistent with the 7-10% that has been published in prior works [2,4]. The supine, mini-open group experienced incision complications in approximately 4% of cases. These differences were not statistically significant, which may be related to the relative rarity of these complications in both groups. One patient in the prone group who experienced a dehiscence required another operation, while no patients in the supine cohort required a return to the operating room. It was not possible to make statistical comparisons on this single event, but it demonstrates the potential gravity of incisional complications.

To date, no published literature has compared operative variables between the prone and supine approaches for the repair of acute Achilles rupture. The observed similarities in average surgical time and blood loss suggest that the supine approach has equal surgical efficiency. If considering the two approaches comprehensively, including the risks associated with placing and maintaining the patient in the prone position, the supine approach may prove to be equally as efficacious with the advantages of an improved cost and safety profile. Postoperatively, PACU time was longer for the prone approach as well, albeit not statistically significant. Any delay in the movement of patients through the PACU can slow operating room proceedings and ultimately delay patient care. 

Current studies evaluating the cost-effectiveness of acute Achilles tendon rupture treatment have primarily focused on comparing operative and non-operative management. Truntzer et al. performed a cost analysis of surgical versus non-surgical treatment of patients undergoing treatment for acute Achilles tendon rupture. This was a database study dependent upon coding for cost analysis, rather than documented costs for specific patients as was utilized for the current study [31]. Additionally, prone versus supine comparison was not done in this study. Other studies have also evaluated the cost-effectiveness of operative versus non-operative treatment, as well as different approaches for acute Achilles tendon rupture [32,33]. However, the approaches assessed have primarily focused on open versus percutaneous procedures rather than supine and prone approaches [32]. It should be noted that these studies were performed outside of the United States, impeding the ability to accurately draw comparisons with U.S. healthcare costs. The current study is the first to assess the cost of prone versus supine acute Achilles tendon repair and demonstrated that the average cost of the prone group exceeded that of the supine group by $1,823. The increased cost in the prone group may be attributable to longer operating room and PACU times. However, further studies are warranted to evaluate the financial burden of specific perioperative variables.

One potential concern with the supine, mini-open approach is that visualization and working space would be limited, leading to greater time in the operating room. However, there was no difference in the surgical time between the two approaches, with a trend toward shorter time with the supine approach. This finding suggests that despite potentially decreased visualization and access to the Achilles tendon, the supine, mini-open technique can be efficiently performed without adding undue complexity.

Another advantage of the surgical technique in the current study is that it was achieved using common surgical techniques and a widely available #2 FiberWire suture. Prior reports of a mini-open surgical approach required specialized instrumentation designed in conjunction with the investigators [6-8]. This may limit the availability of less invasive techniques to certain facilities or increase the cost of performing this type of surgery. The surgical technique described here can be performed at any center with a minimal learning curve.

The study faced several limitations. Many of these were related to the study’s retrospective nature, including the physician’s preference of surgical approach. Additionally, the relatively small sample size may have led to insufficient power to detect certain differences between the two groups. Moving forward, larger prospective studies are needed to better compare prone and supine approaches to repairing the Achilles tendon. Patients in the study were followed for a minimum of six months; this allowed for characterization of many postoperative complications but made it impossible to compare longer-term outcomes. Standard follow-up at the study institution is six months, and patients return on an as-needed basis if any complications arise. Therefore, it can be inferred that patients who did not return did not experience any subsequent complications after six months. Finally, the results of the current study should only be applied to the repair of acute ruptures. Due to the higher degree of tendinosis and retraction seen in chronic ruptures, they may not be amenable to a less invasive technique.

## Conclusions

In conclusion, the mini-open approach in supine position represents a safe option for repairing acute Achilles tendon ruptures with shortened operating room times and similar complication rates, with no patients experiencing re-rupture of the tendon after primary repair, compared to the prone approach. This approach can be utilized in a variety of settings and has been shown to be cost-effective. Larger prospective studies are needed to validate these preliminary results.
